# Herpes Zoster Reactivation After COVID-19 Vaccine with Focus on Postherpetic Neuralgia Prevention: A Case Series

**DOI:** 10.5812/aapm-131366

**Published:** 2023-12-10

**Authors:** Eleni Chrona, Maria Tsoumani, Chrysanthi Batistaki

**Affiliations:** 1 Department of Anaesthesiology, Pain Management Unit, General Hospital of Nikea, Piraeus, Greece; 2 Department of Emergency, Pain Management Unit, General Hospital of Nikea, Piraeus, Greece; 3 Hygeia Private Hospital, Pain Management Unit, Athens, Greece

**Keywords:** Herpes Zoster, Postherpetic Neuralgia

## Abstract

**Introduction:**

Herpes zoster (HZ), also known as shingles, is caused by the reactivation of the varicella-zoster virus (VZV). There have been several reports of HZ associated with COVID-19 vaccination, and the outcomes have varied.

**Case Presentation:**

In this report, we present 4 cases of patients who experienced HZ reactivation after receiving a COVID-19 vaccine. These individuals sought treatment at a pain management center due to postherpetic neuralgia (PHN). While HZ itself can be treated, post-herpetic neuralgia can persist for years, significantly impacting the patients’ quality of life. Therefore, early recognition of this adverse effect is crucial, and patients should receive specialized analgesic support promptly to prevent the development of chronic pain.

## 1. Introduction

Herpes zoster (HZ), also known as shingles, is an infectious disease caused by the reactivation of the varicella-zoster virus (VZV). This virus remains dormant in the dorsal root ganglia and the ganglia of the trigeminal nerve after the initial infection, which typically presents as varicella or chickenpox during childhood (1). Herpes zoster manifests as a painful rash characterized by vesicles followed by erosions along the path of an affected dermatome (1, 2). The rash is invariably painful, typically affecting only one side of the body. One of its most severe complications is postherpetic neuralgia (PHN), which occurs in approximately 60% of patients over the age of 60 years (2). This incidence tends to increase in immunocompromised individuals and with advancing age, underscoring the importance of early identification and treatment of the disease.

Reactivation of HZ, occurring within 1 - 21 days after receiving the COVID-19 vaccine or other vaccines, has been reported (1, 3-8). There is an ongoing debate regarding whether there is a genuine association between different types of COVID-19 vaccines and this clinical phenomenon. Therefore, each newly reported case of such reactivation can contribute to a better understanding of this potential mechanism.

## 2. Case Presentation

We would like to report the cases of 4 patients, all aged over 50, who presented with HZ within 7-20 days after receiving a COVID-19 vaccine targeting SARS-CoV-2 (Table 1). These patients sought treatment at the Pain Management Unit of the General Hospital of Nikea (Ag. Panteleimon) in Piraeus, Greece. They experienced acute, severe pain with evident neuropathic characteristics, as assessed by a visual analog scale (VAS) score exceeding 6 out of 10 during their examination at the pain management unit.

**Table 1.** Demographic Characteristics, Location of Skin Lesions, Time after COVID-19 Vaccination, and Types of Analgesics Used for Treatment

Variables	Age	Location of Herpes Zoster	Time Since COVID-19 Vaccination (Pfizer)	Titrated Pain Medication
**Patient 1**	64	Intercostal neuralgia T7-T8	6 days (after the first dose)	Pregabalin, oxycodone
**Patient 2**	56	Intercostal neuralgia T4-T5	20 days (after the third dose)	Gabapentin, codeine, tramadol, lidocaine TTS
**Patient 3**	65	Cervical neuralgia C5	7 days (after the second dose)	Pregabalin, tramadol, lidocaine TTS
**Patient 4**	65	Occipital neuralgia C2	4 days (after the second dose)	Gabapentin, tramadol

The locations of the HZ and the subsequent pain distribution were as follows: Intercostal neuralgia involving T 7-8 and T 4-5 (patients 1 and 2), cervical root neuralgia at C5 (patient 3), and occipital neuralgia at C2 (patient 4). All patients received a diagnosis from a dermatologist and were prescribed anti-herpes medication, being considered as mild cases of infection. However, the intensity of their pain was severe and required comprehensive management. This included the administration of antiepileptic medications (titrated pregabalin or gabapentin) in combination with transdermal lidocaine. In some cases, small doses of weak opioids were administered for a short duration until symptom control was achieved (Table 1). 

All patients responded positively to the pain treatment, and they continue to be monitored at the pain management unit. Initially, follow-up appointments were scheduled every 2 weeks, which were later adjusted to monthly visits.

## 3. Discussion

Reactivation of HZ within 1-21 days after COVID-19 vaccination has been previously reported, as well as reactivation following various other vaccines, such as yellow fever, hepatitis A, rabies, and influenza A (1). However, there have been reports linking COVID-19 vaccines to such reactivation (1, 3-8), leading to significant debate on this matter. A large cohort study encompassing 1,095,086 vaccinated patients revealed a 0.2% risk of developing shingles (2204 patients) within 60 days after COVID-19 vaccination (all types), while the risk in unvaccinated patients was reported to be 0.11% (1). A potential mechanism proposed for this phenomenon is the alteration of the host’s response to the VZV due to vaccination, particularly involving CD+8 and CD+4 T cell immunity (9). However, a recent systematic review and meta-analysis by Chu et al. failed to establish a clear association between COVID-19 vaccination, an increased incidence of HZ compared to placebo, or differences between various vaccine types (10). These results conflict with the evidence presented in the real-world study by Hertel et al. (1), highlighting the need for further data analysis and interpretation. Additionally, a study by Furer et al. (11) involving 491 patients with autoimmune rheumatological diseases found a prevalence of 1.2% for HZ compared to controls, emphasizing the necessity of additional epidemiological studies on the safety of COVID-19 vaccines in this patient group.

Postherpetic neuralgia is undoubtedly a severe complication characterized by intense neuropathic pain that is resistant to common analgesics. Common features of PHN include mechanical allodynia (pain without a painful stimulus) and thermal hyperalgesia, both related to neuroplasticity (10). The treatment of HZ involves multimodal therapy, including nucleoside analog compounds that inhibit the DNA polymerase of the virus (2). Furthermore, early pain management is of paramount importance, as PHN can sometimes be refractory to treatment (2, 10). There is evidence to suggest that early analgesic support with anticonvulsants and/or antidepressants, which modify nerve pain pathways, in addition to aggressive acute analgesia, can help prevent chronic pain (2). Therefore, prevention and early management are crucial in all cases. While studies may vary in their conclusions regarding the association between COVID-19 vaccination and HZ reactivation, clinician awareness is essential for the prevention and treatment of PHN.

### 3.1. Conclusions

Persistent PHN can be a devastating condition, causing significant distress and a decline in the patient’s quality of life. Therefore, there is a need for heightened awareness among physicians, both in primary care and at vaccination centers, to ensure the early identification of HZ symptoms and to provide patients with proper guidance regarding anti-herpes medication and pain management support at suitable facilities. This proactive approach aims to prevent the development of PHN.

## Data Availability

The dataset presented in this study is available upon request from the corresponding author during submission or after publication. The data are not publicly available due to the fact that they are case series.
